# The item network and domain network of burnout in Chinese nurses

**DOI:** 10.1186/s12912-021-00670-8

**Published:** 2021-08-17

**Authors:** Lin Wu, Lei Ren, Yifei Wang, Kan Zhang, Peng Fang, Xufeng Liu, Qun Yang, Xiuchao Wang, Shengjun Wu, Jiaxi Peng

**Affiliations:** 1 Department of Military Medical Psychology, Air Force Medical University, Xi’an, 710032 China; 2 Tangdu Hospital, Air Force Medical University, Xi’an, 710038 China; 3 College of Teachers, Chengdu University, Chengdu, 610106 China

**Keywords:** Nurse, Mental health, Burnout, Network analysis

## Abstract

**Background:**

As a common social phenomenon, nurses’ occupational burnout has a high incidence rate, which seriously affects their mental health and nursing level. The current assessment mostly uses the total score model and explores the influence of external factors on burnout, while the correlation between burnout items or dimensions is less explored. Ignoring the correlation between the items or dimensions may result in a limited understanding of nurse occupational burnout. This paper explores the item and dimension network structure of the Maslach Burnout Inventory-General Survey (MBI-GS) in Chinese nurses, so as to gain a deeper understanding of this psychological construct and identify potential targets for clinical intervention.

**Methods:**

A total of 493 Chinese nurses were recruited by cluster sampling. All participants were invited to complete the survey on symptoms of burnout. Network analysis was used to investigate the item network of MBI-GS. In addition, community detection was used to explore the communities of MBI-GS, and then network analysis was used to investigate the dimension network of MBI-GS based on the results of community detection. Regularized partial correlation and non-regularized partial correlation were used to describe the association between different nodes of the item network and dimension network, respectively. Expected influence and predictability were used to describe the relative importance and the controllability of nodes in both the item and dimension networks.

**Results:**

In the item network, most of the strongly correlated edges were in the same dimension of emotional exhaustion (E), cynicism (C) and reduced professional efficacy (R), respectively. E5 (Item 5 of emotional exhaustion, the same below) “I feel burned out from my work”, C1 “I have become more callous toward work since I took this job”, and R3 “In my opinion, I am good at my job” had the highest expected influence (z-scores = 0.99, 0.81 and 0.94, respectively), indicating theirs highest importance in the network. E1 “I feel emotionally drained from my work” and E5 had the highest predictability (E1 = 0.74, E5 = 0.74). It shows that these two nodes can be interpreted by their internal neighbors to the greatest extent and have the highest controllability in the network. The spinglass algorithm and walktrap algorithm obtained exactly the same three communities, which are consistent with the original dimensions of MBI-GS. In the dimension network, the emotional exhaustion dimension was closely related to the cynicism dimension (weight = 0.65).

**Conclusions:**

The network model is a useful tool to study burnout in Chinese nurses. This study explores the item and domain network structure of nurse burnout from the network perspective. By calculating the relevant indicators, we found that E5, C1, and R3 were the most central nodes in the item network and cynicism was the central node in the domain network, suggesting that interventions aimed at E5, C1, R3 and cynicism might decrease the overall burnout level of Chinese nurses to the greatest extent. This study provides potential targets and a new way of thinking for the intervention of nurse burnout, which can be explored and verified in clinical practice.

**Supplementary Information:**

The online version contains supplementary material available at 10.1186/s12912-021-00670-8.

## Background

In 2019, the World Health Organization (WHO) recommended for the first time that “burnout” should be included into the International Classification of Disease 11th Edition; thus, indicating that burnout has become a common phenomenon in today’s society and has attracted widespread attention [1]. The term “burnout” was coined by Freudenberger. He believed that burnout is a state of emotional exhaustion that can easily develop during work; i.e., when the work requires a very high level of an individual’s energy and ability, the individual will develop an emotional exhaustion condition [2]. Maslach stated that burnout refers to emotional exhaustion, cynicism, and reduced satisfaction of employees in the professional field of service industry, which includes chronic negative emotions at work and coping with resource depletion in the face of stressors, also known as Burnout Syndrome [3, 4]. With the increasing pressure of medical industry, relatively insufficient proportion of nurses, stressful work, frequent three shifts, high work standards, high level of risks, many emergencies, and long-term high vigilance contribute to the high incidence of burnout [5, 6]. Burnout has a great impact on nurses’ work, which is manifested as decreased enthusiasm for the care of patients (emotional exhaustion) [7], being detached and insensate to the care and strained relationship with patients, intensified conflict with colleagues (cynicism) [8], meaningless nursing work, decline in self-esteem and passive neglect of work (decreased professional efficacy) [9].

Maslach Burnout Inventory-General Survey (MBI-GS) contains a total of 15 items in the three dimensions of emotional exhaustion, cynicism, and reduced personal satisfaction; all of these items are scored from 0 to 6, with 0 representing never and 6 representing very frequent. The score of each dimension is obtained by adding all the items included in each dimension, and the total score of burnout is obtained by adding all the dimensions [10]. The logic behind such a rating model is that each item contributes to burnout to an equal degree (with equal weight). However, there are many different project and dimension combinations that reach a specific total score threshold, and individuals with the same burnout severity may have very different burnout experiences. In addition, this model of equivalence between dimensions and items ignores the relationship between them, which may play a very important role in the development and maintenance of burnout [11]. Some scholars believe that each dimension should be calculated in a weighted manner [12]. In the calculation of the total score, emotional exhaustion accounted for 40%, cynicism and reduced personal satisfaction each accounted for 30% [12]. Some scholars believe that the scores of the three dimensions cannot be added at all; thus, each dimension should be calculated separately and a threshold standard should be established [13, 14].

The network model is an important and innovative method to mathematically analyze and visually display the relationship among complex variables. It is driven by data and is not dependent on prior assumptions of causality among variables [15]. The network model provides an alternative method to conceptualize psychological constructs, which regards psychological constructs as interacting systems, and their components interact with each other, and actively participate in the emergence of this construct rather than the passive indicators of this construct [16, 17]. Taking into consideration the complex nature of burnout, it is reasonable to regard burnout as an interactive system based on this model, which may provide a new perspective to describe and understand burnout. Meanwhile, as compared to mere correlational approaches, network models can also provide several centrality and predictability indicators for each node to quantify their importance and controllability in the entire network [18, 19]. Central variables in a psychological construct may be considered as important intervention targets and may provide a potential target for related interventions. Moreover, community detection can be used to identify communities of nodes where there is a higher density of edges within these communities than between these communities in network models. Recently, an increasing number of studies have used the network model to investigate the network structure for related psychological constructs, including resilience [20], self-worth [21], stigma [22], decision-making competence [23], and personality [24].

This paper attempted to explore the item network of MBI-GS in Chinese nurses, while using community detection to explore communities of MBI-GS from a network perspective, and then to explore the dimension network of MBI-GS based on the results of community detection. In addition, we computed the expected influence and predictability for each item and dimension to quantify their relative importance and controllability in the item network and domain network, respectively. The above results may help us to gain a deeper understanding of burnout and provide some references for relevant interventions. Based on the above, we put forward the following two research hypotheses: first, the nurse burnout network has its unique structure, different items have different importance and connection. Second, three dimensions consistent with previous studies should be found by deconstructing nurse burnout from the perspective of network.

## Methods

### Participants and ethical approval

By using cluster sampling, a total of 493 nurses from several large general hospitals in Shaanxi, China participated in this study (general information shown in Table 1). Inclusion criteria: a. first-line clinical department; b. no history of mental illness; c. be aware of and participate in this experiment voluntarily. Exclusion criteria: a. major life events occur within two weeks; b. use psychotropic drugs within two weeks; c. unwilling to cooperate with this experiment. They completed the questionnaire after signing the written informed consent form. Data were entered by double Person SPSS 24.0 to ensure accuracy and strict confidentiality was maintained. This study was approved by the Ethics Committee of Xijing Hospital (No.KY20183115–1).

**Table 1:** The general situation of the objects (*N* = 472)

Items	Number(n)	Percentage(%)
Sex		
Male	5	1.1
Female	467	98.9
Age (years)		
20–30	417	88.3
31–40	55	11.7
Working age (years)		
0–3	261	55.3
> 3	211	44.7
Diploma		
Junior college	311	65.9
Undergraduate	161	34.1
Marriage		
unmarried	288	61.0
married	184	39.0

### Measures

Maslach Burnout Inventory-General Survey (MBI-GS), jointly developed by American social psychologists Maslach and Jaskson, is a widely used universal scale to measure job Burnout at present [3]. After being authorized by Professor Michael Leiter, who developed the MBI-GS, Chinese scholar Li Chaoping revised the MBI-GS and introduced revised version into China. Firstly, four experts independently translated the English questionnaire into Chinese and discussed the Chinese version. Then six participants were asked to fill in the questionnaire and interview, and some of the words were modified. After that, two English experts were invited to translate the Chinese questionnaire back into English through discussion, and the English version was sent back to professor Michael Leiter. According to Michael’s opinions, the translated questionnaire was partially adjusted and the 16 items of the Chinese version was determined. Then, Li Chaoping’s team carried out exploratory factor analysis on 16 projects of MBI-GS, finding one project of “cynicism” had higher cross load. After deleting the project, the factor analysis is carried out again, and a very ideal result is obtained. The structure of MBI-GS (Chinese version) is completely consistent with that of MBI-GS, which indicates that MBI-GS has good construct validity in China. The revised 15 question version of the Chinese burnout scale has been proved to have good reliability and validity by many studies including clinical medical staff, which meets the psychometric standards [25, 26]. We adopt the revised Chinese version of MBI-GS by scholar Li Chaoping in our study because of the good reliability and validity and more Chinese localization characteristics. The scale contains 15 titles and the following three dimensions: emotional exhaustion (5 items), cynicism (4 items), and reduced personal accomplishment (6 items). Each item is rated on a 7-point scale from 0 (never) to 6 (very frequent). The dimension of occupational efficacy is reverse scored, while the other two dimensions are positively scored. The higher the score, the more serious the burnout [25]. In Li Chaoping’s study, internal consistencies of the three dimensions (emotional exhaustion, cynicism and reduced personal accomplishment) were 0.88, 0.83, and 0.82, respectively [25].

### Network analysis

#### Network estimation and visualization

The item network and domain network were estimated via Gaussian graphical models (GGMs) [27]. GGMs are undirected networks in which the edges represent partial correlations between two nodes after conditioning on all other nodes in the network [28]. In the present study, the GGMs were calculated basing on nonparametric Spearman rho correlation matrices, and the nonparametric Spearman rho correlations among the items and domains are presented in Table S1 and Table S2 (in Supplemental Material). In the item network, the graphical least absolute shrinkage and selection operator (LASSO) algorithm was used to regularize the GGM [28]. This regularization procedure shrinks all edges and sets edges with small partial correlations to zero to obtain a parsimonious and sparse network, which is more stable and easier to interpret [28]. Meanwhile, the tuning parameter was set to 0.5 to adequately balance the sensitivity and specificity of picking out true edges [29]. Due to low variables (3 dimensions) but high samples (472 individuals), we used the unregularized model selection approach rather than regularization techniques to estimate the dimension network [30]. Visualizations of the item and domain networks were derived from the Fruchterman–Reingold algorithm, which locate nodes with stronger and numerous connections near the center of the network and weakly associated nodes on the periphery [31]. In the visualized networks, blue edges represent positive correlations and red edges represent negative correlations. Thicker edges indicate stronger correlations between the nodes.

#### Centrality and predictability analysis

Recent studies have shown that strength is the most reliable centrality index. The other centrality indices, such as betweenness and closeness, seem especially unsuitable for assessing the importance of nodes in psychological networks [32, 33]. Node strength is the sum of the absolute value of the edge weights attached to a node, and it may misinterpret the actual effect of nodes on the remaining network when there are negative edge weights in the network [34, 35]. Thus, we calculated the expected influence for both the item and dimension networks. This measure has replaced node strength in the most recent studies due to the evidence that it effectively considers both positive and negative edges within the network [35, 36]. Higher expected influence values indicate greater importance in the network [22]. The estimation, visualization, and centrality index of the item and dimension networks were carried out in the R-package *qgraph* [37]. In addition, we computed the predictability of each node in the item and dimension networks by using the R-package *mgm* [19]. Predictability is defined as the variance of a node, which is explained by all of its neighboring nodes, and this index could characterize the controllability of the network [19].

#### Community detection

The spinglass algorithm was used to identify communities of items in the item network. It is based on the principle that the edges should connect nodes of the same community, whereas nodes belonging to different communities should not be connected [38]. It is important to note that an item can only be part of one community by using this procedure. Since the spinglass algorithm can yield different results in the same sample, we assessed the stability of the solution by running the algorithm 100 times and extracted the number of communities with the highest frequency. To complement the results, we also used the walktrap algorithm, which is based on the principle that adjacent nodes tend to belong to the same community [38]. This algorithm was shown to have high accuracy in simulation studies and was used in empirical network papers [39, 40].

#### Network accuracy and stability

We examined the robustness of the item and dimension networks by using the R-package *bootnet* [41, 42]. First, the accuracies of edge weights were evaluated by calculating the 95% confidence intervals (CI) using a non-parametric bootstrap approach (2000 bootstrap samples). Second, the stabilities of nodes’ expected influences were evaluated by computing the correlation stability (CS) coefficient, using a case-dropping bootstrap approach. The value of the CS coefficient should not be less than 0.25 and preferably should be more than 0.5 [42]. Third, bootstrapped difference tests (2000 bootstrap samples and α = 0.05) for edge weights and nodes’ expected influences were performed to evaluate whether two edge weights or two nodes’ expected influences differ significantly from each other.

## Results

### Demographic descriptive statistical analysis

A total of 493 questionnaires were sent out and all of them were recovered, excluding several unanswered questions and obvious perfunctory answers to the questionnaire; 472 questionnaires were recovered effectively and the effective recovery rate was 95.74%. The age of participants ranged from 20 years to 39 years, with a mean of 26.24 years (SD = 3.61).

The general situation of the participants is shown in Table 1.

### Descriptive statistical analysis and network analysis of Maslach burnout inventory-general survey (MBI-GS) projects

Some parameters of descriptive statistical analysis and network analysis of the MBI-GS items are shown in Table 2. In this study, internal consistencies were 0.93, 0.88, 0.91 respectively of the three dimensions and 0.87 of the whole items.

**Table 2:** Some parameters of descriptive statistical analysis and network analysis of Maslach Burnout Inventory-General Survey (MBI-GS) dimensions and items

Dimensions and items	M	SD	EI^a^	Pre
Emotional exhaustion (E)	11.98	6.11	0.19	0.56
1.I feel emotionally drained from my work (E1)	2.61	1.26	−0.03	0.74
2.I feel used up at the end of the day (E2)	2.75	1.34	−0.19	0.70
3.I feel tired when I get up in the morning and have to face another day at work (E3)	2.40	1.46	0.27	0.67
4.Working with people all day is a real strain for me (E4)	2.42	1.40	−0.27	0.68
5.I feel burned out from my work (E5)	1.80	1.48	0.99	0.74
Cynicism (C)	6.53	4.83	0.89	0.58
6.I have become more callous toward work since I took this job (C1)	1.72	1.43	0.81	0.72
7.I have become less enthusiastic about my work (C2)	1.68	1.39	0.74	0.71
8.I doubt the significance of my work (C3)	1.53	1.40	0.47	0.64
9.I have become more and more indifferent in the contribution of my job (C4)	1.60	1.40	−1.74	0.42
Reduce professional efficacy (R)	14.55	7.55	−1.08	0.05
10.I deal effectively with the problems of clients (R1)	2.50	1.46	−2.53	0.38
11.I feel that I am contributing to my company (R2)	2.60	1.60	−0.69	0.54
12.In my opinion, I am good at my job (R3)	2.40	1.50	0.94	0.71
13.I feel very happy when I accomplish some tasks of my job (R4)	2.20	1.51	0.32	0.69
14.I have accomplished many worthwhile things in this job (R5)	2.65	1.50	0.65	0.71
15.I am confident that I can accomplish all tasks effectively (R6)	2.19	1.51	0.27	0.68

Abbreviations: M, mean; SD, standard deviation; EI, expected influence; Pred, predictability; E (Emo): Emotional exhaustion; C (Cyn): Cynicism; R (Eff): Reduce professional efficacy

^a^ z-scores rather than raw centrality indices

### Network analysis

#### Item network

**Fig. 1:**
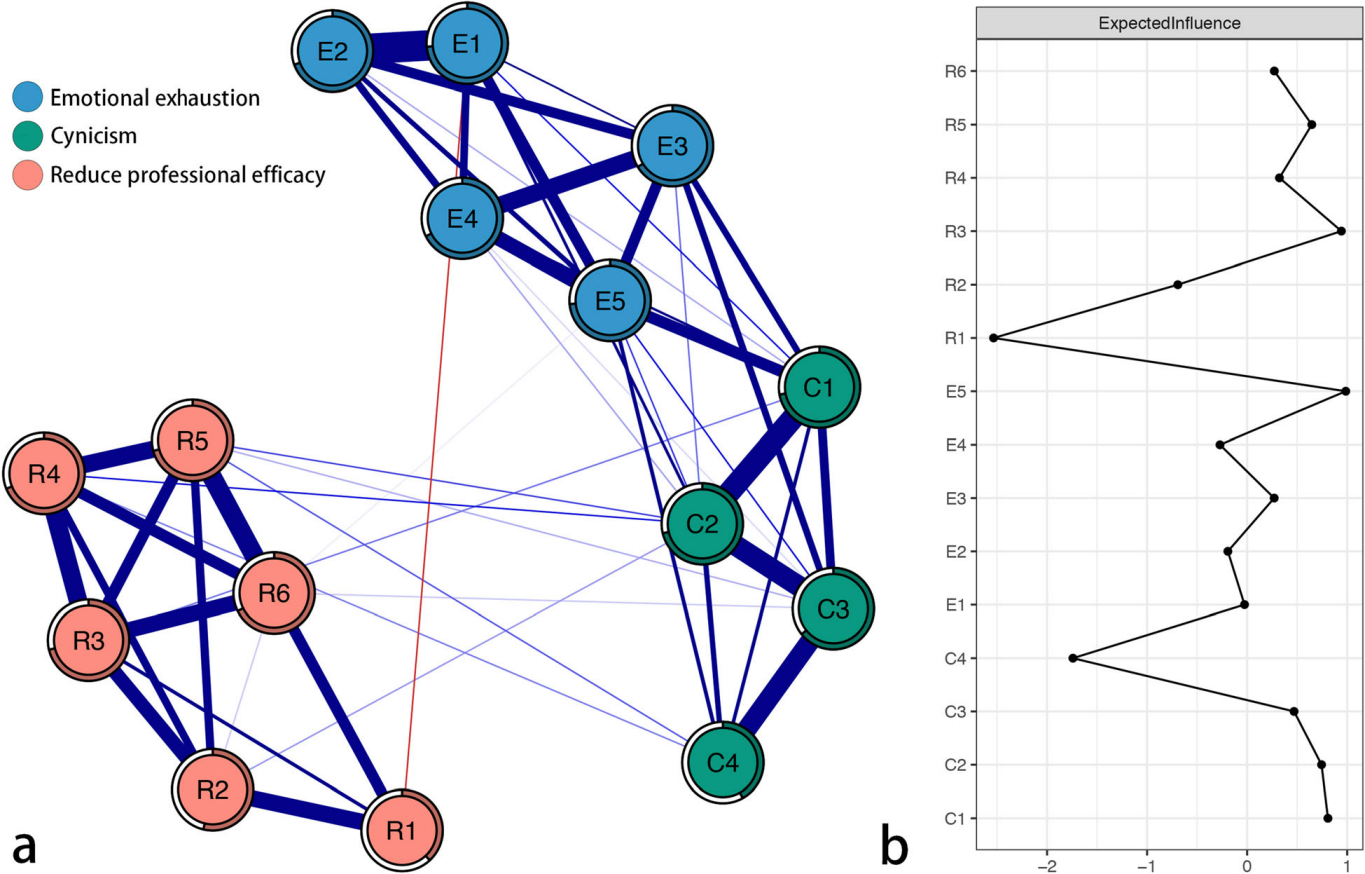
Item network of the Maslach Burnout Inventory (MBI-GS) in Chinese nurses. Note: (**a**) Blue edges represent positive correlations, and red edges represent negative correlations. The thickness of the edge reflects the magnitude of the correlation. The rings around nodes depict their predictability. (**b**) Centrality plot depicting the expected influence of each item in the network (z-score). E1 = I feel emotionally drained from my work; E2 = I feel used up at the end of the day; E3 = I feel tired when I get up in the morning and have to face another day at work; E4 = Working with people all day is a real strain for me; E5 = I feel burned out from my work; C1 = I have become more callous toward work since I took this job; C2 = I have become less enthusiastic about my work; C3 = I doubt the significance of my work; C4 = I have become more and more indifferent in the contribution of my job; R1 = I deal effectively with the problems of clients; R2 = I feel that I am contributing to my company; R3 = In my opinion, I am good at my job; R4 = I feel very happy when I accomplish some tasks of my job; R5 = I have accomplished many worthwhile things in this job; and R6 = I am confident that I can accomplish all tasks effectively

The item network is shown in Fig. 1a. There were several obvious characteristics in this network. First, 53 (50.5%) among the 105 possible edges did not have zero values and all these edges had a positive value except for the one edge between E1 “I feel emotionally drained from my work” and R1 “I deal effectively with the problems of clients” (weight = − 0.04). Second, twelve edges with strongest regularized partial correlations existed between E1 “I feel emotionally drained from my work” and E2 “I feel used up at the end of the day” (weight = 0.45), between C1 “I have become more callous toward work since I took this job” and C2 “I have become less enthusiastic about my work” (weight = 0.33), between C2 “I have become less enthusiastic about my work” and C3 “I doubt the significance of my work” (weight = 0.31), between C3 “I doubt the significance of my work” and C4 “I have become more and more indifferent in the contribution of my job” (weight = 0.30), between R5 “I have accomplished many worthwhile things in this job” and R6 “I am confident that I can accomplish all tasks effectively” (weight = 0.29), between R3 “In my opinion, I am good at my job” and R4 “I feel very happy when I accomplish some tasks of my job” (weight = 0.28), between E3 “I feel tired when I get up in the morning and have to face another day at work” and E4 “Working with people all day is a real strain for me” (weight = 0.26), between R4 “I feel very happy when I accomplish some tasks of my job” and R5 “I have accomplished many worthwhile things in this job” (weight = 0.26), between R1 “I deal effectively with the problems of clients” and R2 “I feel that I am contributing to my company” (weight = 0.25), between R3 “In my opinion, I am good at my job” and R6 “I am confident that I can accomplish all tasks effectively” (weight = 0.24), between E4 “Working with people all day is a real strain for me” and E5 “I feel burned out from my work” (weight = 0.23), and between R2 “I feel that I am contributing to my company” and R3 “In my opinion, I am good at my job” (weight = 0.22). Third, node predictability is shown as a ring around the node in Fig. 1a. The value of node predictability ranged from 0.38 to 0.74 (see Table 2), with an average value of 0.64. This means that on average, 64% of the variance of the node can be explained by its neighbors in the item network. E1 “I feel emotionally drained from my work” (predictability = 0.74) and E5 “I feel burned out from my work” (predictability = 0.74) had the highest predictability: 74% of their variance can be explained by their neighboring nodes. R1 “I deal effectively with the problems of clients” (predictability = 0.38) had the lowest predictability: 38% of its variance can be explained by its neighboring nodes.

The z-score value of the expected influence for each node was computed to assess its relative importance in the item network (see Fig. 1b and Table 2). Three nodes with the highest expected influence were E5 “I feel burned out from my work” (z-scores = 0.99), R3 “In my opinion, I am good at my job” (z-scores = 0.94), and C1 “I have become more callous toward work since I took this job” (z-scores = 0.81). This indicates that these three nodes are the most associated nodes in the present network from a statistical point of view. The node with the lowest expected influence was R1 “I deal effectively with the problems of clients” (z-scores = − 2.53), which indicates that this node is the least associated node in the present network from a statistical point of view.

Bootstrap 95% confidence interval indicated that the estimation of edge weights was accurate (Fig. S1 in the Supplementary Material). In addition, the CS coefficient of nodes’ expected influences was 0.75, indicating that the estimation of nodes’ expected influence was adequately stable (Fig. S2 in the Supplementary Material). The bootstrapped difference test for edge weights showed that in the item network, a moderate proportion of the differences among edge weights was significant (Fig. S3 in the Supplementary Material). Moreover, the bootstrapped difference tests for nodes’ expected influences showed that in the item network, a small to moderate proportion of the differences among nodes’ expected influences was significant (Fig. S4 in the Supplementary Material).

The spinglass algorithm identified a mean of three communities of items corresponding to the three dimensions of MBI-GS, as proposed originally and confirmed by Maslach and her colleagues [3]. One community comprised items E1, E2, E3, E4, and E5, forming the dimension of “Emotional exhaustion”. One community was formed by items C1, C2, C3, and C4, and all of them constituted the dimension of “Cynicism”. The other community was formed by items R1, R2, R3, R4, R5, and R6 and was represented the dimension of “Reduced professional efficacy”. Meanwhile, the walktrap algorithm identified the same three communities as the spinglass algorithm.

#### Dimension network

**Fig. 2:**
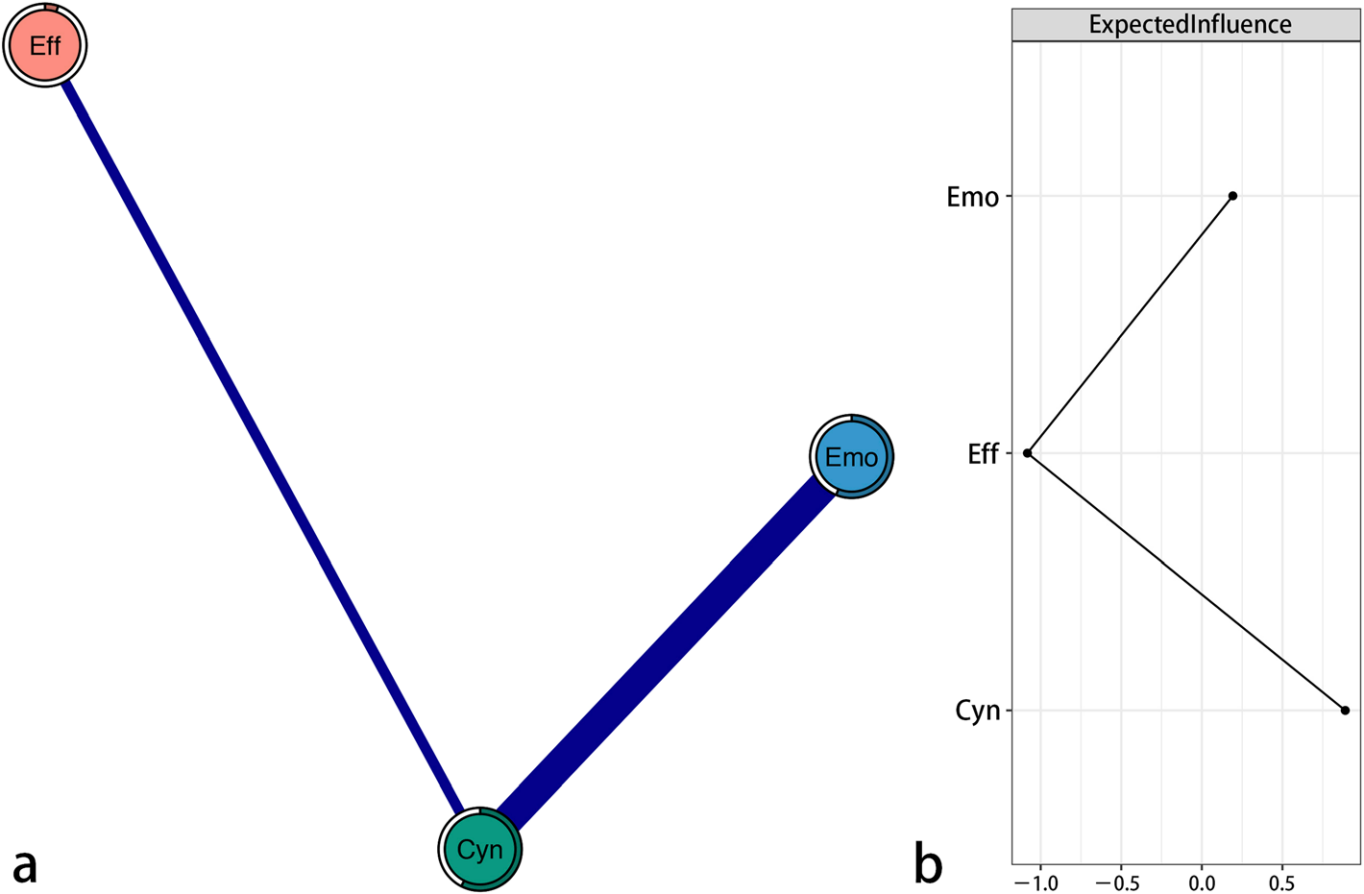
Domain network of the Maslach Burnout Inventory (MBI-GS) in Chinese nurses. Note: (**a**) Blue edges represent positive correlations, and red edges represent negative correlations. The thickness of the edge reflects the magnitude of the correlation. The rings around nodes depict their predictability. (**b**) Centrality plot depicting the expected influence of each domain in the network (z-score). Emo = Emotional exhaustion; Cyn = Cynicism; Eff = Reduced professional efficacy

The dimension network is shown in Fig. 2a. There were several obvious characteristics in the network. First, 2 (66.7%) edges among the 3 possible edges did not have zero values and these two edges had positive values. Second, the edge between “Emotional exhaustion” and “Cynicism” (weight = 0.65) had strong unregularized partial correlation, while the edge between “Cynicism” and “Reduced professional efficacy” (weight = 0.23) had relatively weak unregularized partial correlation. There was no unregularized partial correlation between “Emotional exhaustion” and “Reduced professional efficacy”. Third, node predictability is shown as a ring around the node in Fig. 2a. The node predictability of “Emotional exhaustion”, “Cynicism”, and “Reduced professional efficacy” was 0.56, 0.58, and 0.05 (see Table 2), respectively. This means that 56% of the variance of “Emotional exhaustion”, 58% of the variance of “Cynicism”, and 5% of the variance of “Reduced professional efficacy” can be explained by its neighbors in the dimension network.

The z-score value of the expected influence for each node was computed to assess its relative importance in the dimension network (see Fig. 2b and Table 2). “Cynicism” had the highest expected influence (z-scores = 0.89), which indicates that this node is the most associated node in the dimension network from a statistical point of view.

Bootstrap 95% confidence interval indicated that the estimation of edge weights was accurate (Fig. S5 in the Supplementary Material). In addition, the CS coefficient of nodes’ expected influences was 0.75, indicating that the estimations of node’s expected influence were adequately stable (Fig. S6 in the Supplementary Material). The bootstrapped difference test for edge weights showed that in the dimension network, difference between two edge weights was significant (Fig. S7 in the Supplementary Material). Moreover, the bootstrapped difference tests for nodes’ expected influences showed that in the dimension network, all differences among nodes’ expected influences were significant (Figs. S8 in the Supplementary Material).

## Discussion

Previous studies used the network analysis to explore the relationship between each dimension of burnout and other diseases or symptoms, but ignored the internal relationship of each item and dimension of burnout [43, 44]. This study is a supplement to the relationship among the items and dimensions of burnout. In this study, the network model was applied for the first time to explore the item network of MBI-GS in Chinese nurses. In addition, community detection was used to explore communities of MBI-GS, and then we used network analysis to investigate the dimension network of MBI-GS based on the results of community detection. Three communities, which are consistent with the original three dimensions as proposed originally and confirmed by Maslach and her colleagues, were obtained through the spinglass algorithm and the walktrap algorithm. The results verify the three-dimension theory of burnout from the network perspective. In the item network and dimension network, we evaluated the expected influence and predictability for each item and dimension to quantify their relative importance and controllability. These two networks provide some insights into gaining an understanding of nurse burnout and potential targets for interventions.

Burnout critically affects the physical and mental health of nurses and the quality of nursing, resulting in the loss of nurses, the decline of nursing quality and increased unsatisfaction in patients [45]. Nurses’ job burnout is serious due to their personal, management, organization, work and other reasons [9, 46]. Accumulating evidence indicate that burnout is alarming prevalent among Chinese nurses in the both overall and three dimensions [47–49], especially those who are working in the ICU setting [50]. Studies have also found that nurses are more serious in the dimension of emotional exhaustion, doctors are more serious in the dimension of cynicism, while other medical staff (medical practitioners other than doctors and nurses, such as laboratory technician, pharmacist, anesthesiologist, etc.) have more serious decline in professional efficacy [51, 52].

### Item network

The item network structure showed that the strongest edges were within each dimension. Three edges with strongest regularization partial correlation were between E1“I feel emotionally drained from my work” and E2 “I feel used up at the end of the day” (the emotional exhaustion dimension), between C1 “I have become more callous toward work since I took this job” and C2 “I have become less enthusiastic about my work” (the cynicism dimension), and between R5 “I have accomplished many worthwhile things in this job” and R6 “I am confident that I can accomplish all tasks effectively” (the reduced professional efficacy dimension). The strong regularization partial correlation between two nodes indicates that these two nodes have high co-occurrence. E1 “I feel emotionally drained from my work” and E2 “I feel used up at the end of the day” are similar statements and may describe the same aspect. Both of them describe the state of depression and lack of enthusiasm caused by work. Besides, drained and used up have a similar meaning while describing emotion. Thus, there is a strong correlation between them. From a theoretical perspective, for C1 “I have become more callous toward work since I took this job” and C2 “I have become less enthusiastic about my work”, loss of interest may make individuals unwilling to pay a lot of time and energy to their work to a large extent, i.e., they are no longer enthusiastic about their work subjectively. For R5 “I have accomplished many worthwhile things in this job” and R6 “I am confident that I can accomplish all tasks effectively”, achieving valuable work may be a reflection of one’s ability and can also be seen as a positive feedback, which increases psychological capital and effectively improves one’s confidence in work. Accordingly, when a person is very confident at his work, he may be able to or mobilize resources to perform a good job. Previous research has also illustrated this point [53, 54]. In the cross-dimensional edges, E5 “I feel burned out from my work” and C1 “I have become more callous toward work since I took this job” also have a positive correlation, which may play a “bridge” role in connecting these two dimensions. There were many other items that were not related to each other. For example, the items of the emotional exhaustion dimension were less connected with the items of the reduced occupational efficacy dimension. However, there were relatively close links between the items of the cynicism dimension and the items of the emotional exhaustion dimension.

Network analysis could help us examine the relative importance of each item in the resilience network, and nodes with higher centrality may have greater influences on the network than nodes with lower centrality. As mentioned in a previous study, “in the absence of any other clinical information, if we have to choose a clinical feature as the target of intervention, selecting the most central node may be a feasible heuristic method” [55]. Thus, central items may be considered as targets for the related intervention [34, 56, 57]. In the item network, three nodes with the highest expected influence were E5 “I feel burned out from my work” (the emotional exhaustion dimension), R3 “In my opinion, I am good at my job” (the reduced professional efficacy dimension) and C1 “I have become more callous toward work since I took this job” (the cynicism dimension). This means that these three nodes may play the most important roles in activating and maintaining the present network. Interventions on E5 “I feel burned out from my work”, C1 “I have become more callous toward work since I took this job”, and R3 “In my opinion, I am good at my job” may transfer to E4 “Working with people all day is a real strain for me”, C2 “I have become less enthusiastic about my work”, and R4 “I feel very happy when I accomplish some tasks of my job”, respectively, and then transfer to more nodes. This will have an effect on each dimension, and then affect the whole network. This suggests that the intervention for the above three nodes may transfer the effect to other nodes to reduce other symptoms indirectly, so as to reduce the overall level of nurses’ job burnout quickly and effectively, providing us with new insights on the intervention of nurses’ job burnout and a reference for the selection of potential intervention targets [16, 58].

The predictability results showed that on average, 64% of the variance of nodes in the item network can be explained by their neighbors, indicating that the item network was more likely to be self-determined. The predictabilities of E1 “I feel emotionally drained from my work” and E5 “I feel burned out from my work” were 74%, indicating that the two nodes were greatly influenced by their neighboring nodes. This finding suggests that we could control E1 “I feel emotionally drained from my work” and E5 “I feel burned out from my work” by intervening them or their strong neighbors, rather than via other variables that are not included in the network, such as environmental and biological factors [19, 59]. In particular, it should be noted that predictability is the upper bound estimation because the direction of the edge is unknown in a cross-sectional study [19].

### Dimension network

According to the spinglass algorithm and walktarp algorithm, burnout can be divided into three communities. Excitingly, these three communities exactly correspond to the original dimension division and item composition of MBI-GS. These results also verify the three-dimensional theory of burnout from the network perspective. There was a strong unregularized partial correlation between the emotional exhaustion dimension and cynicism dimension, which indicated that these two dimensions have high co-occurrence.

The cynicism dimension had a relatively small correlation with the reduced professional efficacy dimension, while the emotional exhaustion dimension had no correlation with the reduced professional efficacy dimension. The cynicism dimension had the highest expected influence and was associated with the other two dimensions, suggesting that clinical intervention in this dimension might yield the greatest benefit. The above results indicate that the cynicism dimension is more central in burnout and has become the core dimension of burnout. The findings contradict with the previous research results, which suggested that emotional exhaustion was the central dimension. Previous studies have shown that when the score of the emotional exhaustion dimension is considered as the dependent variable, cynicism is introduced into the equation and it occupies an important position. The higher the degree of cynicism, the higher the degree of emotional exhaustion [60]. Shirom also holds the same view, and he states that as displayed by the Maslach’s three-dimensional burnout scale, only the dimension of emotional exhaustion is necessary, while the other two dimensions are auxiliary. Cynicism is a form of reflection of an individual in the state of emotional exhaustion, while the decrease in reduced professional efficacy can be regarded as continuation of emotional exhaustion [61]. In addition, the cynicism dimension had the highest predictability, indicating that about 60% of its variance can be explained by the other two dimensions. Both the item network and the dimensional network showed that emotional exhaustion had minimal correlation with reduced professional efficacy. We considered that the emotional exhaustion dimension and reduced professional efficacy dimension might not be directly related, and the connection between these two dimensions might be transmitted through the cynicism dimension. This finding still needs to be assessed further.

There are several limitations to the present study. First, this study was a cross-sectional study and it could not determine the direction of the edge in the network. Thus, the causal relationship between nodes could not be obtained. Time series data can be used to explore the temporal causality between nodes in future studies. Second, the network in this study estimated between-subject effects on a group level. Thus, it is possible that characteristics, such as centrality and network structure, may not remain the same on an individual level. Third, the network structure was limited by the nodes in the network; thus, there may be some burnout aspects that were not included in the present network. In addition, different scales of burnout may have different characteristics of the network structure, which can be further explored in future studies. Fourth, some of the items were questioned as being redundant due to their similarity in expression, such as E1 “I feel emotionally drained from my work” and E2 “I feel used up at the end of the day”, which interfered with the expected influence and predictability of our results.

## Conclusions

This study reconstructs nurses’ job burnout from the network perspective, finding its unique network characteristics and providing potential targets for in-depth understanding and intervention of nurses’ job burnout. Future studies should include the demographic data of the subjects and continue to explore the network characteristics of burnout of all kinds of subjects. Time series data will be used to find the time causal relationship between nodes. Moreover, the intervention targets suggested in this study should be verified in the further clinical practice.

## Supplementary Information

**Additional file 1: Table S1.** Nonparametric Spearman rho correlation matrix of the items of the MBI-GS. **Table S2.** Nonparametric Spearman rho correlation matrix of the domains of the MBI-GS. **Fig. S1.** Accuracy of edge weights. **Fig. S2.** Stability of node expected influences. **Fig. S3.** Bootstrapped difference test for edge weights. **Fig. S4.** Bootstrapped difference test for node expected influences. **Fig. S5.** Accuracy of edge weights. **Fig. S6.** Stability of node expected influences. **Fig. S7.** Bootstrapped difference test for edge weights. **Fig. S8.** Bootstrapped difference test for node expected influences.

## Data Availability

The datasets used and/or analysed during the current study are available from the corresponding author on reasonable request.

## Abbreviations

MBI-GS: Maslach Burnout Inventory-General Survey
WHO: World Health Organization
ICU: Intensive care unit
GGMs: Gaussian graphical models
LASSO: Least absolute shrinkage and selection operator
CI: Confidence intervals
CS: Correlation stability
M: Mean
SD: Standard deviation
EI: Expected influence
Pred: Predictability
E (Emo): Emotional exhaustion
C (Cyn): Cynicism
R (Eff): Reduce professional efficacy
E1: I feel emotionally drained from my work
E2: I feel used up at the end of the day
E3: I feel tired when I get up in the morning and have to face another day at work
E4: Working with people all day is a real strain for me
E5: I feel burned out from my work
C1: I have become more callous toward work since I took this job
C2: I have become less enthusiastic about my work
C3: I doubt the significance of my work
C4: I have become more and more indifferent in the contribution of my job
R1: I deal effectively with the problems of clients
R2: I feel that I am contributing to my company
R3: In my opinion, I am good at my job
R4: I feel very happy when I accomplish some tasks of my job
R5: I have accomplished many worthwhile things in this job
R6: I am confident that I can accomplish all tasks effectively
