# Supplementary material for: The item network and domain network of burnout in Chinese nurses
Source: BMC Nurs. 2021 Aug 17;20:147. doi: 10.1186/s12912-021-00670-8 (PMC8369754; doi:10.1186/s12912-021-00670-8)
Supplement: Supplementary file 1 — Additional file 1: Table S1. Nonparametric Spearman rho correlation matrix of the items of the MBI-GS. Table S2. Nonparametric Spearman rho correlation matrix of the domains of the MBI-GS. Fig. S1. Accuracy of edge weights. Fig. S2. Stability of node expected influences. Fig. S3. Bootstrapped difference test for edge weights. Fig. S4. Bootstrapped difference test for node expected influences. Fig. S5. Accuracy of edge weights. Fig. S6. Stability of node expected influences. Fig. S7. Bootstrapped difference test for edge weights. Fig. S8. Bootstrapped difference test for node expected influences. [file 12912_2021_670_MOESM1_ESM.docx]

**The item network and domain network of burnout in Chinese nurses**

Lin Wu^1,†^, Lei Ren^1,†^, Yifei Wang^1^, Kan Zhang^2^, Peng Fang^1^, Xufeng Liu^1^, Qun Yang^1^,

Xiuchao Wang^1^, Shengjun Wu^1,*^, Jiaxi Peng^3,*^

^1^ Department of Military Medical Psychology, Air Force Medical University,

710032, Xi’an, China

^2^ Tangdu Hospital, Air Force Medical University, 710038, Xi’an, China

^3^ College of Teachers, Chengdu University, 610106, Chengdu, China

^*^ Correspondence: wushj@fmmu.edu.cn; pjx_cdu@163.com

^†^ Lin Wu and Lei Ren contributed equally to this work

**Supplementary Materials**

*Note:*

E(Emo)=Emotional exhaustion;

C(Cyn)=Cynicism;

R(Eff)=Reduce professional efficacy;

E1 =I feel emotionally drained from my work;

E2 =I feel used up at the end of the day;

E3 =I feel tired when I get up in the morning and have to face another day at work;

E4 =Working with people all day is a real strain for me;

E5 =I feel burned out from my work;

C1 =I have become more callous toward work since I took this job;

C2 =I have become less enthusiastic about my work;

C3 =I doubt the significance of my work;

C4=I have become more and more indifferent in the contribution of my job;

R1 =I deal effectively with the problems of clients;

R2 =I feel that I am contributing to my company;

R3 =In my opinion, I am good at my job;

R4 =I feel very happy when I accomplish some tasks of my job;

R5 =I have accomplished many worthwhile things in this job;

R6 =I am confident that I can accomplish all tasks effectively.

**Item network:**

1. Table S1. Nonparametric Spearman rho correlation matrix of the items of the MBI-GS

2. Figure S1. Accuracy of edge weights

5. Figure S2. Stability of node expected influences

3. Figure S3. Bootstrapped difference test for edge weights

4. Figure S4. Bootstrapped difference test for node expected influences

|  | E1 | E2 | E3 | E4 | E5 | C1 | C2 | C3 | C4 | R1 | R2 | R3 | R4 | R5 | R6 |
| --- | --- | --- | --- | --- | --- | --- | --- | --- | --- | --- | --- | --- | --- | --- | --- |
| E1 | 1.000 |  |  |  |  |  |  |  |  |  |  |  |  |  |  |
| E2 | 0.756** | 1.000 |  |  |  |  |  |  |  |  |  |  |  |  |  |
| E3 | 0.618** | 0.638** | 1.000 |  |  |  |  |  |  |  |  |  |  |  |  |
| E4 | 0.638** | 0.630** | 0.692** | 1.000 |  |  |  |  |  |  |  |  |  |  |  |
| E5 | 0.670** | 0.636** | 0.689** | 0.694** | 1.000 |  |  |  |  |  |  |  |  |  |  |
| C1 | 0.559** | 0.526** | 0.624** | 0.577** | 0.668** | 1.000 |  |  |  |  |  |  |  |  |  |
| C2 | 0.512** | 0.453** | 0.552** | 0.506** | 0.581** | 0.726** | 1.000 |  |  |  |  |  |  |  |  |
| C3 | 0.469** | 0.402** | 0.562** | 0.488** | 0.564** | 0.657** | 0.707** | 1.000 |  |  |  |  |  |  |  |
| C4 | 0.327** | 0.308** | 0.393** | 0.337** | 0.472** | 0.523** | 0.544** | 0.615** | 1.000 |  |  |  |  |  |  |
| R1 | -0.069 | -0.005 | 0.021 | 0.030 | 0.048 | 0.024 | 0.089 | 0.042 | 0.057 | 1.000 |  |  |  |  |  |
| R2 | 0.002 | 0.008 | 0.081 | 0.067 | 0.111* | 0.162** | 0.244** | 0.195** | 0.198** | 0.525** | 1.000 |  |  |  |  |
| R3 | 0.098* | 0.067 | 0.137** | 0.145** | 0.165** | 0.250** | 0.257** | 0.233** | 0.218** | 0.484** | 0.635** | 1.000 |  |  |  |
| R4 | 0.060 | 0.056 | 0.113* | 0.102* | 0.211** | 0.249** | 0.305** | 0.239** | 0.253** | 0.420** | 0.589** | 0.726** | 1.000 |  |  |
| R5 | 0.080 | 0.076 | 0.151** | 0.105* | 0.158** | 0.259** | 0.307** | 0.272** | 0.261** | 0.445** | 0.591** | 0.704** | 0.715** | 1.000 |  |
| R6 | 0.062 | 0.053 | 0.164** | 0.131** | 0.188** | 0.228** | 0.249** | 0.235** | 0.177** | 0.505** | 0.545** | 0.706** | 0.679** | 0.715** | 1.000 |

Table S1. Nonparametric Spearman rho correlation matrix of the items of the MBI-GS.

^*^ *p* < 0.05;^**^ *p* < 0.01


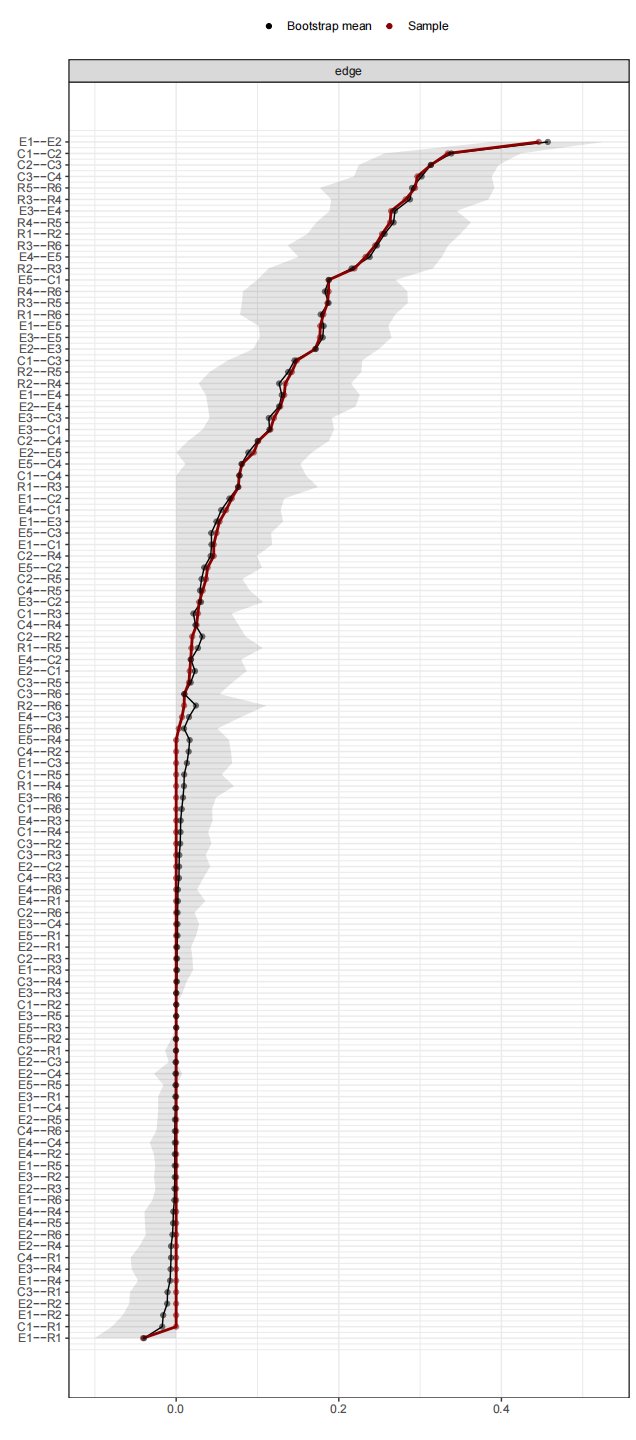


Figure S1. Accuracy of edge weights

*Note*: The red line depicts the sample edge weights and the gray bar depicts the bootstrapped confidence interval.


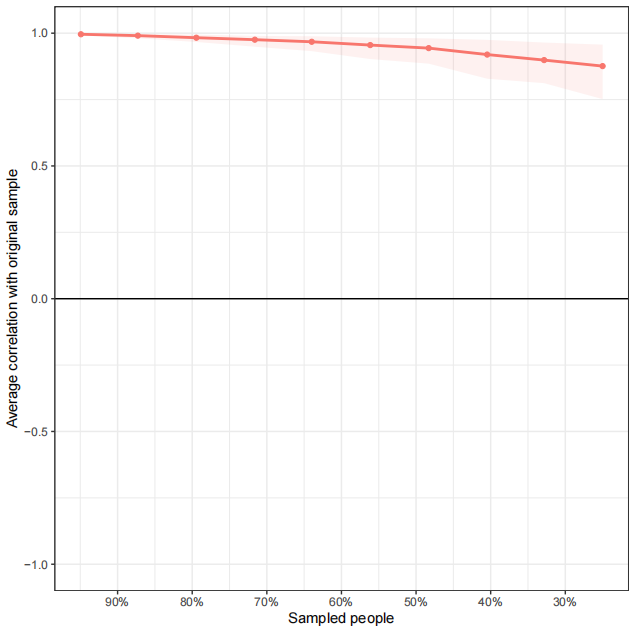


Figure S2. Stability of node expected influences

*Note*: The red bar represents the average correlation between expected influences in the full sample and subsample with the red area depicting the 2.5th quantile to the 97.5th quantile.


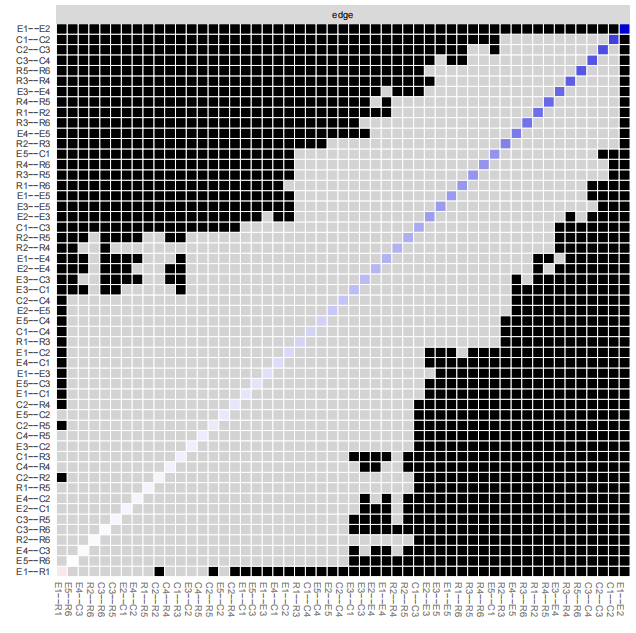


Figure S3. Bootstrapped difference test for edge weights

Note: Gray boxes indicate edge weights that do not differ significantly from one another, while black boxes indicate edge weights that do differ significantly. Blue and red boxes on the diagonal correspond to edge weights with positive and negative correlations, respectively.


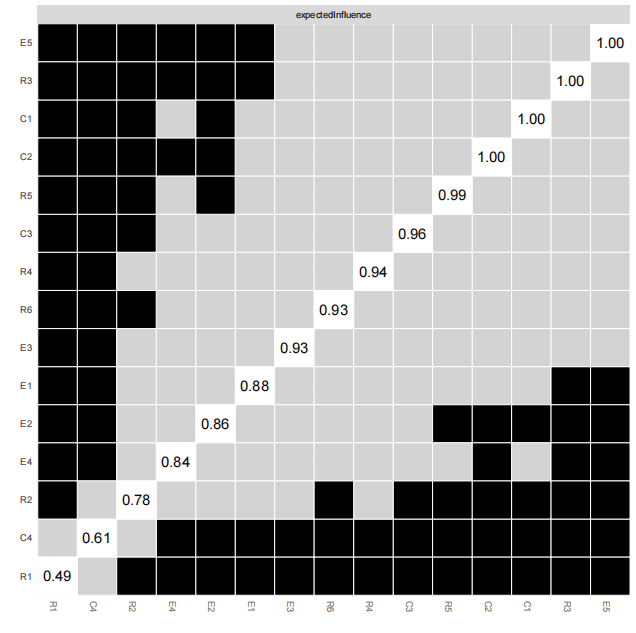


Figure S4. Bootstrapped difference test for node expected influences

*Note*: Gray boxes indicate node expected influences that do not differ significantly from one another, while black boxes indicate node expected influences that do differ significantly. The number in the white boxes (i.e., diagonal line) represent the value of node expected influences.

**Domain network:**

1. Table S2. Nonparametric Spearman rho correlation matrix of the domains of the MBI-GS

2. Figure S5. Accuracy of edge weights

5. Figure S6. Stability of node expected influences

3. Figure S7. Bootstrapped difference test for edge weights

4. Figure S8. Bootstrapped difference test for node expected influences

Table S2. Nonparametric Spearman rho correlation matrix of the domains of the MBI-GS.

|  | Emo | Cyn | Eff |
| --- | --- | --- | --- |
| Emo | 1.000 |  |  |
| Cyn | 0.671** | 1.000 |  |
| Eff | 0.144** | 0.305** | 1.000 |

^*^ *p* < 0.05;^**^ *p* < 0.01

**
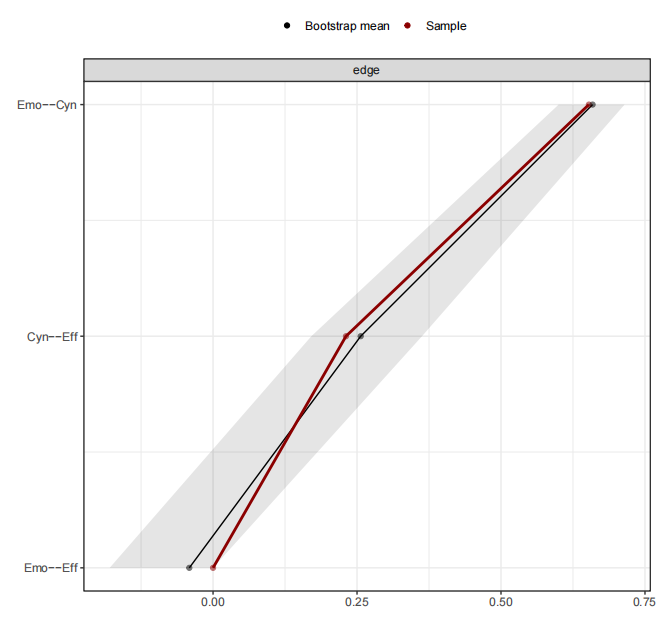
**

Figure S5. Accuracy of edge weights

*Note*: The red line depicts the sample edge weights and the gray bar depicts the bootstrapped confidence interval.

**
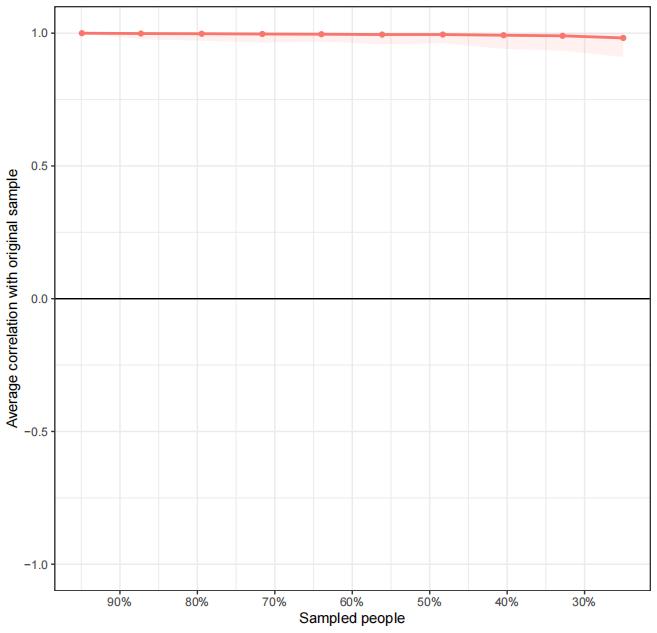
**

Figure S6. Stability of node expected influences

*Note*: The red bar represents the average correlation between expected influences in the full sample and subsample with the red area depicting the 2.5th quantile to the 97.5th quantile.

**
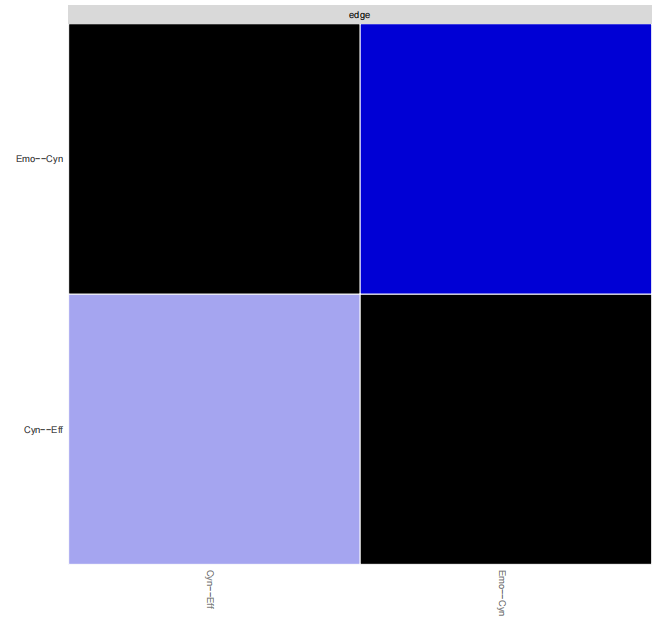
**

Figure S7. Bootstrapped difference test for edge weights

*Note:* Gray boxes indicate edge weights that do not differ significantly from one another, while black boxes indicate edge weights that do differ significantly. Blue and red boxes on the diagonal correspond to edge weights with positive and negative correlations, respectively.

**
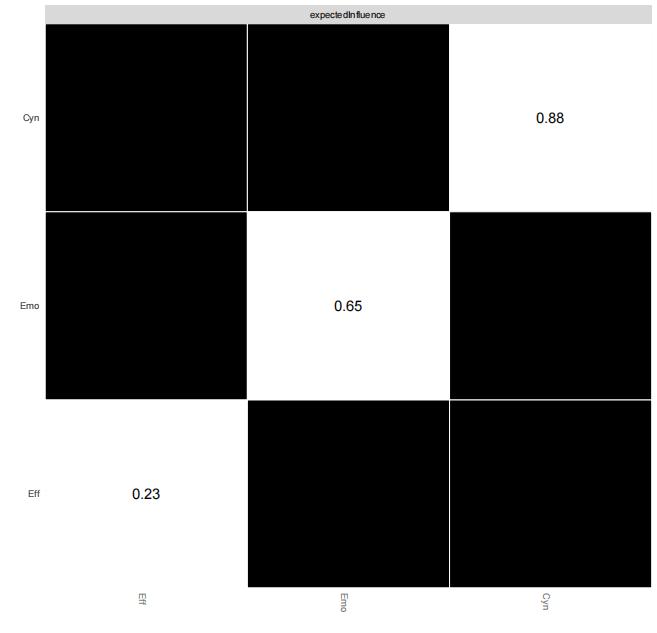
**

Figure S8. Bootstrapped difference test for node expected influences

*Note*: Gray boxes indicate node expected influences that do not differ significantly from one another, while black boxes indicate node expected influences that do differ significantly. The number in the white boxes (i.e., diagonal line) represent the value of node expected influences.
